# Ineffectiveness of tirzepatide in mitigating Doxorubicin-induced oxidative stress and cognitive deficits in a rat model

**DOI:** 10.3389/fphar.2025.1638527

**Published:** 2025-08-05

**Authors:** Salma A. Alolayan, Ahmad H. Alhowail

**Affiliations:** Department of Pharmacology and Toxicology, College of Pharmacy, Qassim, University, Buraydah, Saudi Arabia

**Keywords:** DXN, tir, cognitive impairment, chemotherapy, oxidative stress, antioxidants, mitochondria

## Abstract

Doxorubicin (DXN) is widely utilized for the treatment of various cancer types. However, prolonged DXN administration induces vascular impairment and neurological effects, including cognitive deficits. Here, we investigated the efficacy of tirzepatide (TIR), an antidiabetic agent that improve cognitive function in diabetic rats, in alleviating cognitive impairment in DXN-treated rats. Forty female Wistar rats were allocated to four experimental groups: Control, DXN (5 mg/kg body weight (BW)), TIR (1.35 mg/kg BW), and DXN + TIR (5 mg DXN/kg BW followed by 1.35 mg TIR/kg BW). The compounds were injected intraperitoneally over four cycles. Mortality rates, alterations in body weight (BW), behavior, and oxidative stress markers were evaluated. The mortality rates in the control and TIR groups remained 0%, whereas those in the DXN and DXN + TIR groups were 30% and 40%, respectively. A decline in body weight was detected in rats treated with DXN and DXN + TIR, significantly relative to those in the control and TIR groups. Behavioral assessments revealed that subjects administered with DXN showed impairments, as evidenced by their performance in the Y-maze, MORT, and EPMT tasks. Furthermore, the addition of TIR did not alleviate these impairments. Superoxide dismutase (SOD) and glutathione peroxidase (GPx) levels were diminished in the DXN and DXN + TIR groups although not changes in catalase, whereas reactive oxygen species (ROS), lipid peroxidation, and malondialdehyde (MDA) levels were increased relative to those in the control group. Additionally, the functionality of mitochondrial complex-I was found to be compromised in DXN and DXN + TIR in comparison to the control group. In conclusion, the findings demonstrate that DXN induces neurotoxicity and cognitive impairments through the mechanism of increased oxidative stress. Furthermore, the concurrent administration of TIR did not mitigate the neurotoxic effects, as evidenced by persistent oxidative stress and cognitive deficits.

## Graphical Abstract

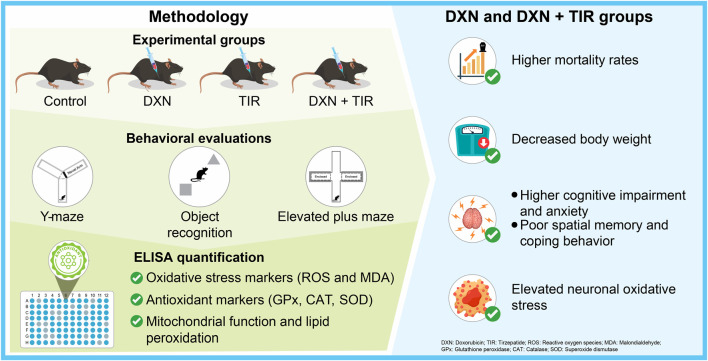

## 1 Introduction

Cancer fundamentally stems from the disruption of normal cellular growth and division, highlighting the necessity for effective therapeutic strategies to address this disease (Zhang et al., 2022). Recent estimates reveal that there are approximately 16 million cancer survivors in the United States, with this number expected to increase to 22 million by 2030 (Miller et al., 2019). A range of alternative therapies is currently available for cancer treatment. Historically, surgery has been the most established and preferred method for cancer treatment, while radiation therapy was first employed to treat localized cancers in the 1960s (Khan et al., 2019). However, interventions such as surgery and radiation alone are inadequate for comprehensive cancer management (Chen and Kuo, 2017). Contemporary cancer treatment modalities encompass immunotherapies, chemotherapeutic agents, and biological substances. Despite these advancements, a standardized treatment for metastatic cancer that significantly reduces mortality rates and extends survival remains elusive (Liu et al., 2024).

Chemotherapeutic agents used for cancer treatment are either developed from antibiotics or chemically synthesized (Anand et al., 2023). Chemotherapy is used as a supplementary treatment to surgery and radiation for hindering cell division and triggering apoptosis (DeVita and Chu, 2008). However, it is commonly associated with adverse effects that may vary depending on the type of cancer (Rana et al., 2017). Conventional treatments may result in a variety of side effects, including infection-induced leukopenia and anemia (Jameus et al., 2021). The most prevalent negative effects of chemotherapy are reduced oxygen supply to the cells and alopecia. Furthermore, throughout the treatment, patients may experience other symptoms, such as nausea, vomiting, lack of appetite, hormonal fluctuations, sleep disturbances, alterations in taste, and changes in skin color (Rana et al., 2017; Matsos et al., 2017). Alterations in cognitive function, often described as “chemobrain,” is a frequent consequence of chemotherapy, characterized by difficulties related to memory, attention, and reaction speed (Matsos et al., 2017).

Doxorubicin (DXN) is a widely used chemotherapeutic agent, which is antibiotic, belonging to the anthracycline group, is produced by *Streptomyces peucetius* and is effective in treating breast and ovarian cancers, soft tissue and bone sarcomas, thyroid cancers, and bladder cancers (Trendowski, 2015). It causes the separation of DNA strands and by inserting itself between DNA base pairs, it disrupts DNA and RNA synthesis, thereby stalling the movement of topoisomerase II, leading to DNA damage and initiation of apoptosis (Johnson-Arbor et al., 2017). Long-term use of this substance causes vascular damage and has neurological consequences (Al-Abbasi et al., 2016; Rizk et al., 2017). It also induces cardiotoxicity and gastrointestinal complications (Tacar et al., 2013). Several studies have linked DXN treatment with neurobiological effects such as cognitive impairment (Alhowail and Aldubayan, 2023; Alsikhan et al., 2023). Patients with breast cancer, administered doxorubicin and cyclophosphamide, have increased susceptibility to cognitive impairment (Ramalho et al., 2017).

Glucagon-Like Peptide-1 (GLP-1) is a hormone integral to the regulation of insulin and glucose metabolism, and it is utilized in the treatment of type 2 diabetes and obesity (Zheng et al., 2024). Beyond its metabolic functions, GLP-1 plays a crucial role in the brain, particularly within the hippocampus, which is essential for learning, memory, and neurogenesis (Diz-Chaves et al., 2022). The mechanism of action of GLP-1 includes the enhancement of insulin secretion, the suppression of glucagon release, the delay of gastric emptying, and the reduction of appetite (Zheng et al., 2024). The GLP-1 agonist TZP has been shown to alleviate cognitive dysfunction caused by diabetes in rats by reducing neuroinflammation, as evidenced by decreased levels of TNF-α, IL-6, IL-1β, and phosphorylated NF-κB, thereby mitigating insulin resistance (Cheng et al., 2022). Moreover, recent preclinical and experimental research has examined the interaction between GLP-1 analogs and doxorubicin for their cardioprotective effects in animal models (Wilson et al., 2020). The cardioprotective mechanism proposed involves the enhancement of mitochondrial function, the reduction of oxidative stress, the attenuation of inflammation, and the inhibition of apoptosis (Luna-Marco et al., 2023; Nuamnaichati et al., 2020; Atef et al., 2023).

Glucagon-like peptide-1 receptors (GLP-1Rs) are located in the hippocampus, dorsomedian, paraventricular, and arcuate regions of the brain (Baggio and Drucker, 2014; Cabou and Burcelin, 2011). Neurons in the caudal portion of the nucleus of the solitary tract synthesize pre-proglucagon and are regarded as the principal source of GLP-1 in the brain, with projections targeting GLP-1Rs (Zheng et al., 2024). Recent studies demonstrate a strong correlation between GLP-1 agonists and the treatment of neurodegenerative diseases by reducing amyloid-beta and tau pathology in the hippocampus, thereby making them a promising target for Alzheimer’s disease treatment (Hölscher, 2014; Kopp et al., 2022). Although the etiology of neurodegenerative diseases is not fully elucidated, the inflammatory response is a critical factor in the progression of pathology (Gilhus and Deuschl, 2019). Tirzepatide (TIR) is classified as a GLP-1 and glucose-dependent insulinotropic polypeptide (GIP) receptor agonist (Jastreboff et al., 2022). TIRe demonstrates significant efficacy in reducing glucose levels and promoting weight loss (Chaplin, 2023). Tirzepatide has been revealed to mitigate oxidative stress by decreasing reactive oxygen species (ROS) and malondialdehyde (MDA) levels, while simultaneously enhancing mitochondrial function in the liver, muscle, and potentially the brain (Luna-Marco et al., 2024). Chemotherapeutic agents such as doxorubicin can induce neuroinflammation and oxidative stress, leading to neuronal damage and cognitive decline (Alhowail, 2024). However, no studies have yet identified the protective effect of TIR in mitigating the impact of DXN on oxidative stress and cognitive function.

Despite the fact that chemobrain is frequently observed in breast cancer patients and DXN is a widely administered anticancer in their treatment protocols, there is a significant gap in research concerning the protective effects of tirzepatide on DXN-induced memory impairments in female rats, as well as the exploration of potential intervention methods. Therefore, this research investigated innovative therapeutic strategies for preventing and managing chemotherapy-induced cognitive impairment and oxidative stress, commonly known as chemobrain. The study evaluated the neuroprotective effects of TIR in mitigating cognitive deficits and oxidative stress in DXN-administered rats.

## 2 Materials and methods

### 2.1 Drugs

DXN injection (2 mg/mL) was attained from EBEWE Pharma GmbH Nfg KG (A-4866; Unterach, Austria) and the TIR injection (5 mg/0.5 mL) was obtained from Eli Lilly Italy (Sesto Fiorentino 50019, Italy).

### 2.2 Animals

Forty female Wistar rats weighing between 163 and 198 g and aged between 10–12 weeks were attained from the College of Pharmacy’s Animal Centre at Qassim University. The rats were boarded in propylene cages with five numbers per cage and reserved in a controlled environment (25°C ± 2°C; 12-h light-dark cycle). They were allowed with *ad libitum* access to food and water. All animals were observed every other day to measure their BW and mortality rate, and behavioral tests were accomplished through the light cycle. The ethics was permitted by the deanship for Graduate Studies and Scientific Research at Qassim University (Approval # 23-69-07), and followed to the NIH Procedures for the Care and Use of Laboratory Animals for all animals utilized in the experiments.

### 2.3 Drug treatment schedule

A cohort of 40 female rats was randomly allocated into four diverse groups, each consisting of 10 rats: Control, DXN, TIR, and DXN + TIR. The DXN group was administered an intraperitoneal dose of 5 mg/kg body weight (BW) for four cycles (cumulative dose 20 mg/kg), with each cycle separated by a three-day interval, and this dosage was consistent with the therapeutic levels typically administered to individuals receiving cancer treatment (Alsaud et al., 2023; Zhong et al., 2023; Alharbi et al., 2020). The TIR group received an intraperitoneal dose of 1.35 mg/kg BW for four cycles, expanding upon prior research that identified the neuroprotective properties of the administered dose in diabetic rats (Guo et al., 2023). The combination group was treated with both DXN (5 mg/kg) and TIR (1.35 mg/kg BW) via intraperitoneal injection. The Control group received an intraperitoneal injection of saline. One day post-treatment, all subjects underwent a series of behavioral assessments, including the Y-maze, Elevated Plus Maze (EPM), and Object Recognition Test (ORT), to evaluate cognitive function. Following these assessments, the animals were euthanized, and their brains were harvested for biochemical analysis.

### 2.4 Mortality rate and body weight

The daily routine monitoring of mortality rates provided vital insights for the ongoing research. The cages underwent cleaning every 2 days, and deceased animals were promptly removed. Regular assessment of body weight was crucial for evaluating general health status. Thus, body weight was measured every 3 days, enabling the detection of subtle variations and assisting in the early identification of potential health concerns.

### 2.5 Behavioral assessments

Behavioral assessments frequently reflect the functional status of specific brain regions. In the present study, the Y-maze, Novel Object Recognition (NOR), and Elevated Plus Maze (EPM) tests were utilized. The Y-maze test is particularly dependent on the hippocampus, especially the CA1 and CA3 subregions, as well as inputs from the prefrontal cortex. This test evaluates spontaneous alternation behavior, an inherent tendency in rodents to explore a new arm rather than returning to a previously visited one (Kraeuter et al., 2019). The NOR test primarily relies on the perirhinal cortex, which is influenced by the hippocampus and synaptic plasticity, and is based on the natural preference of rodents to investigate novel objects over familiar ones (Antunes and Biala, 2011). The EPM is a well-established paradigm for assessing anxiety-like behavior in rodents, involving specific neural circuits, neurotransmitters, and brain regions (Lezak et al., 2022). Key regions include the amygdala, which processes sensory input related to threats, the prefrontal cortex, and the ventral hippocampus, which is involved in processing contextual information (Lezak et al., 2022; Gaspar et al., 2023).

### 2.6 Y-maze test

This test is a widely utilized method for assessing short-term working spatial learning and memory in animals. The Y-maze is composed of three wooden arms, each with dimensions of 50 cm in length, 10 cm in width, and 17 cm in height, designed to enhance visibility. These arms are placed on the floor with lighting evenly distributed above. Initially, one of the three arms is blocked, restricting the animals to two arms during a 15-min training session. Following this, the animals are allowed to explore all three arms, including the previously inaccessible arm, during a 5-min testing session. A 3-h interval separates the training session from the test. The test is recorded on video to analyze the frequency of entries into each arm and the duration spent in the novel arm (the arm that was initially closed). The evaluation of the frequency of entries into the novel arm, the total entries into all arms, and the time spent in the novel arm serves as an indicator of memory function (Alotayk, 2023).

### 2.7 Novel-Object Recognition Test

The test, formulated by Ennaceur and Delacour, evaluates the duration animals require to exhibit a preference for exploring unfamiliar objects over familiar ones (Ennaceur and Delacour, 1988). The experimental apparatus consisted of a wooden brown open box (41 cm × 41 cm × 41 cm) with two identical objects (a small black jewelry box) positioned equidistantly from the center. During the training session, animals were placed at the center and allowed to explore the objects for 15 min. Following this, one object was substituted with a different one (a small white/black jewelry box). A 3-h rest interval was observed between the training session and the test. The test was recorded on video to ascertain the time spent by the animals in investigating the novel object. This time was subsequently calculated and analyzed to evaluate memory function.

### 2.8 Elevated plus maze test

The EPM test was implemented to investigate anxiety-like behavior in rodents, following the protocol delineated by Handley and Mithani (Guo et al., 2023). The apparatus featured four identical arms, each measuring 50 cm by 10 cm, with two arms open and two enclosed, configured in a plus shape. The maze was elevated 50 cm above the ground for the duration of the experiment. Testing occurred in a quiet, dimly lit setting, and the apparatus was thoroughly cleaned between trials to eliminate any scent cues. At the start of each session, the animals were positioned at the center of the maze, facing an open arm. Behavioral activity was observed for 5 min to assess the frequency of entries into, and the duration spent within, both the open and closed arms (Naz et al., 2022).

### 2.9 Collection of the brain tissue samples

Rats were euthanized through CO_2_ asphyxiation within a glass chamber (Conlee et al., 2005). Following this, the brains were extracted and thoroughly washed with chilled phosphate-buffered saline to remove any blood. The brain tissue was subsequently homogenized in phosphate-buffered saline with the addition of a protease inhibitor, utilizing sonication (Q-sonica homogenizer, 35 Hz pulses for 15 s). The homogenate was then subjected to centrifugation for 10 min at 12,000 × g. Finally, the brain tissue lysate was preserved at −80°C.

### 2.10 Enzyme-linked Immunosorbent assays

Brain tissues were harvested from all animals and subsequently homogenized using a neuronal lysis buffer. Protein concentrations were measured via the BCA assay, followed by the analysis of specific protein levels through sandwich ELISA. This analysis included oxidative stress biomarkers such as ROS (cat. No. RK15283) and MDA (cat. No. RK15281), alongside antioxidant enzymes including SOD (cat. No. RK07054), GPx (cat. No. RK03696), and catalase (cat. No. RK03551). The assays were conducted on the brain homogenate using rat ELISA kits, all sourced from ABclonal Technology, Woburn, MA, United States, in conformity with the manufacturer’s instructions.

### 2.11 Investigation of complex I mitochondria activity

The quantification of brain samples was performed using the Bradford method. The mitochondrial function in oxidizing NADH was evaluated through spectrophotometric analysis at 340 nm. Results were reported as the quantity of NADH oxidized per milligram of protein and as a percentage in comparison to the control group (Alhowail et al., 2022).

### 2.12 Investigation of lipid peroxidation

Initially, the brain tissues were prepared and homogenized using the N-PER™ lysis buffer. Following this, the total protein content of each sample was determined through the bicinchoninic acid assay, which was a prerequisite for the subsequent lipid peroxidation assay. The levels of lipid peroxidation were evaluated using a spectrophotometric technique involving thiobarbituric acid. To estimate the lipid peroxidation index, the production of thiobarbituric acid-reactive substances (TBARS) was measured at a wavelength of 532 nm. This TBARS measurement was then normalized by dividing it by the total protein content, yielding the amount of TBARS produced per milligram of protein (Alhowail, 2024).

### 2.13 Statistical analysis

Data were analyzed via software provided by GraphPad Prism form 10.4 and displayed as the mean ± standard error of the mean (SEM). Statistical analysis for each group was performed with a one-way ANOVA, tailed by Tukey’s post-hoc test. A p-value of ≤ 0.05 was deliberated statistically varied.

## 3 Results

### 3.1 Effect of DXN and TIR on mortality in rats

**FIGURE 1:**
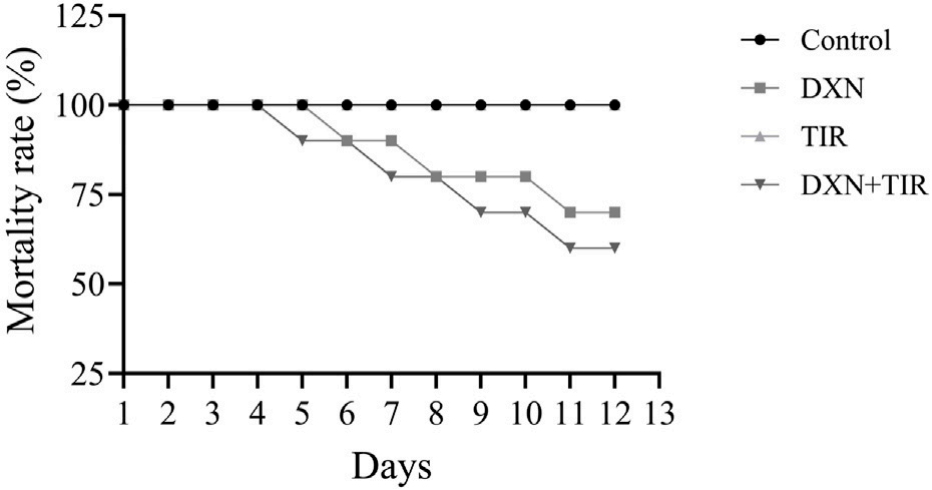
Effect of doxorubicin (DXN) and tirzepatide (TIR) on the mortality rate in different groups. Data are characterized as mean ± SEM (n = 10 for each group).

**FIGURE 2:**
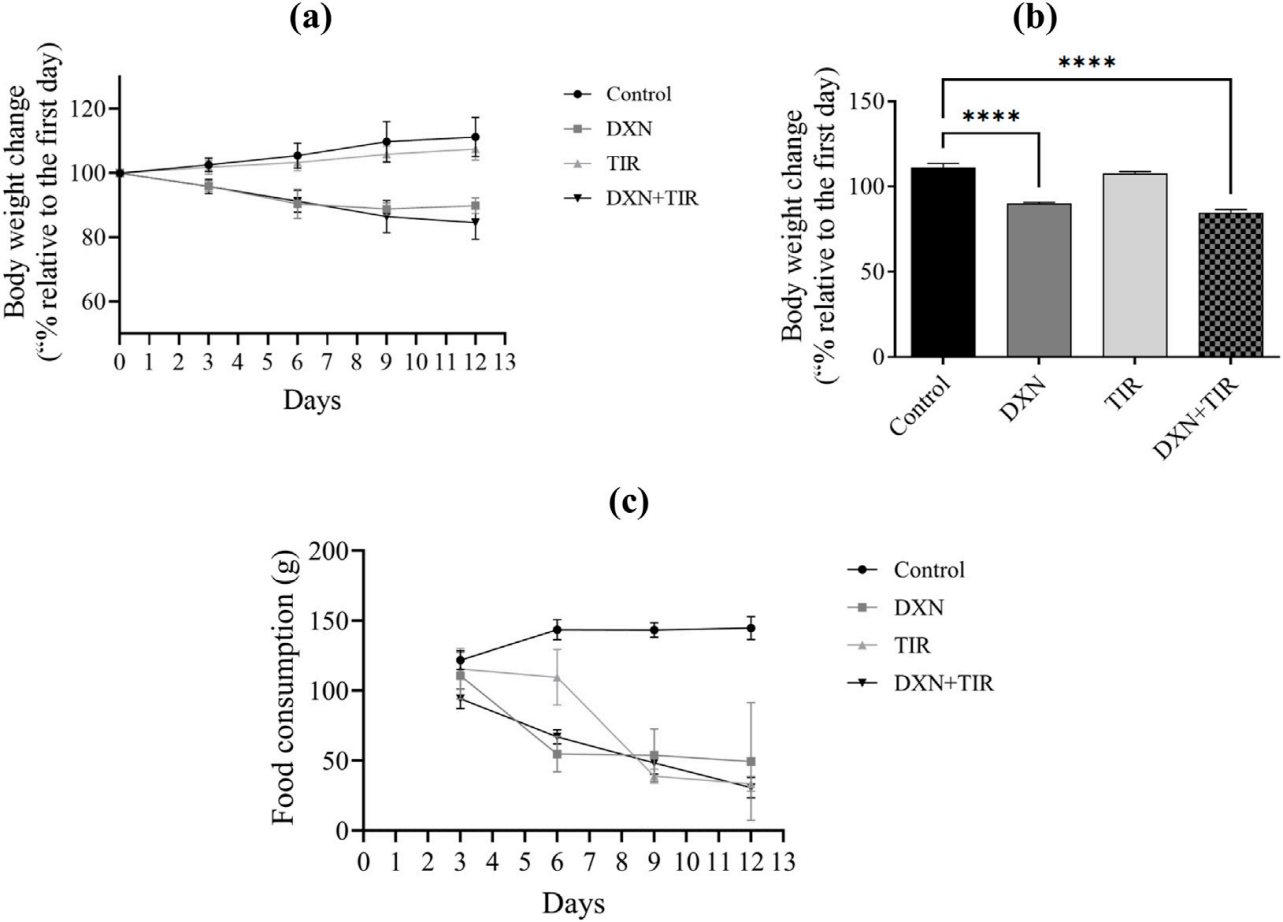
Effect of doxorubicin (DXN) and tirzepatide (TIR) on body weight and food intake. **(a,b)** The body weight showed percentage change compared to baseline (Day 1). **(c)** The average food intake per animal per day. Data are presented as mean ± SEM (n = 6/group). ****p > 0.0001.

The therapy schedule and experimental design are illustrated in Figure 1. Following 2 weeks of treatment, mortality rates in both the control and TIR groups remained at 0%. In the DXN and combination treatment (DXN + TIR), the mortality rate increased to 20% on the eighth day. However, the mortality rates in the DXN and combination treatment (DXN + TIR) groups were 30% and 40%, respectively, on day 12, which was the end of the study (Figure 2).

### 3.2 Effect of DXN and TIR on body weight and food consumption

After 2 weeks of treatment, the weight of animals increased only in the control group. A partial reduction in body weight was noted for the TIR group. However, the body weight was significantly reduced in both DXN and DXN + TIR groups compared to the control (Figures 2a,b). In addition, the food intake data revealed that DXN, TIR alone, and TIR combined with DXN reduced the average of food intake related to the control (Figure 2c).

### 3.3 Y-maze performance following DXN and TIR treatment

**FIGURE 3:**
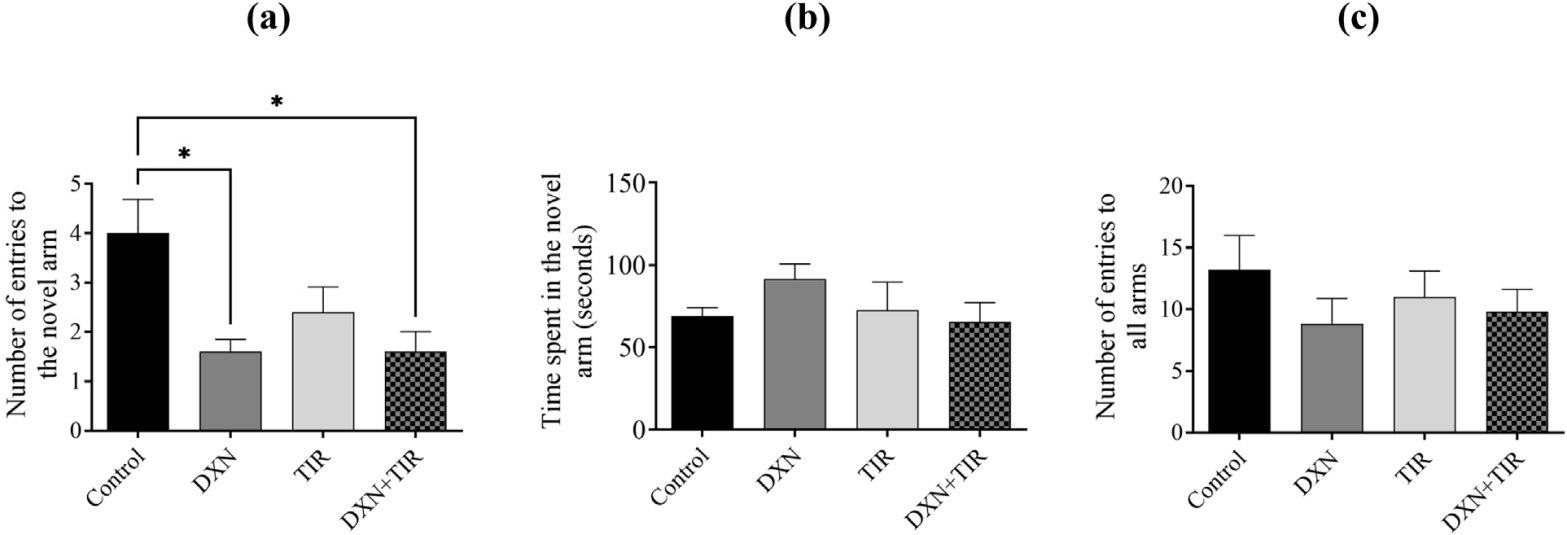
The effect of tirzepatide (TIR) on doxorubicin (DXN)-induced behavioral changes in rats was evaluated using a Y-maze task. **(a)** Effect of DXN and TIR on the total sum of entries into the novel arm of the Y-maze. **(b)** The Effect of DXN and TIR on the duration spent in the novel arm of the Y-maze. **(c)** Overall sum of entries into all arms. Alterations were deemed significant at *p < 0.05, related to the control group (n = 6).

The DXN and DXN + TIR treatment groups showed a statistically significant decrease (*p* < 0.05) in the number of new arm entries when compared to the control group (Figure 3a). During the Y-maze task, the DXN group recorded the longest time spent in the new arm (Figure 3b). The total sum of entries was evaluated to ensure the animals’ ability to move was not impaired, revealing no significant differences (Figure 3c).

### 3.4 Novel-Object Recognition Test (NORT) performance following DXN and TIR treatment

**FIGURE 4:**
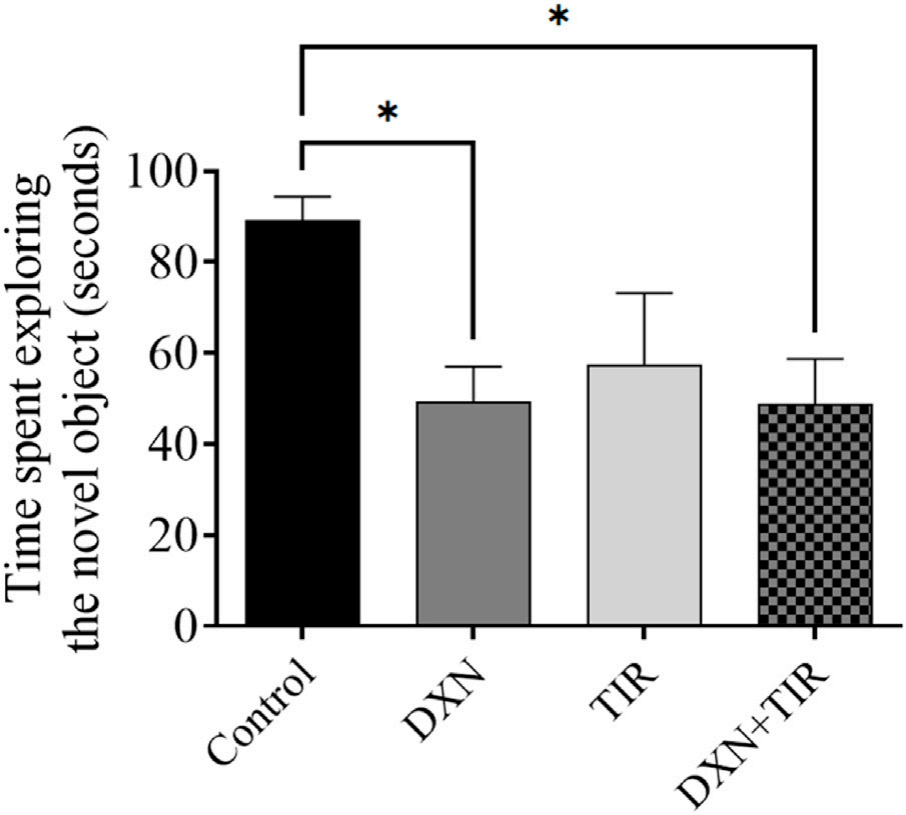
Effect of tirzepatide (TIR) treatment on doxorubicin (DXN)-induced changes in the behavior of rats assessed using NORT. Modifications were considered substantial at *p < 0.05 related to the control group (n = 6).

In the NORT (Figure 4), the control group showed a statistically substantial increase in the time consumed exploring the new object related with that in the DXN and DXN + TIR groups (*p* < 0.05).

### 3.5 Elevated Plus Maze test (EPMT) performance following DXN and TIR treatment

**FIGURE 5:**
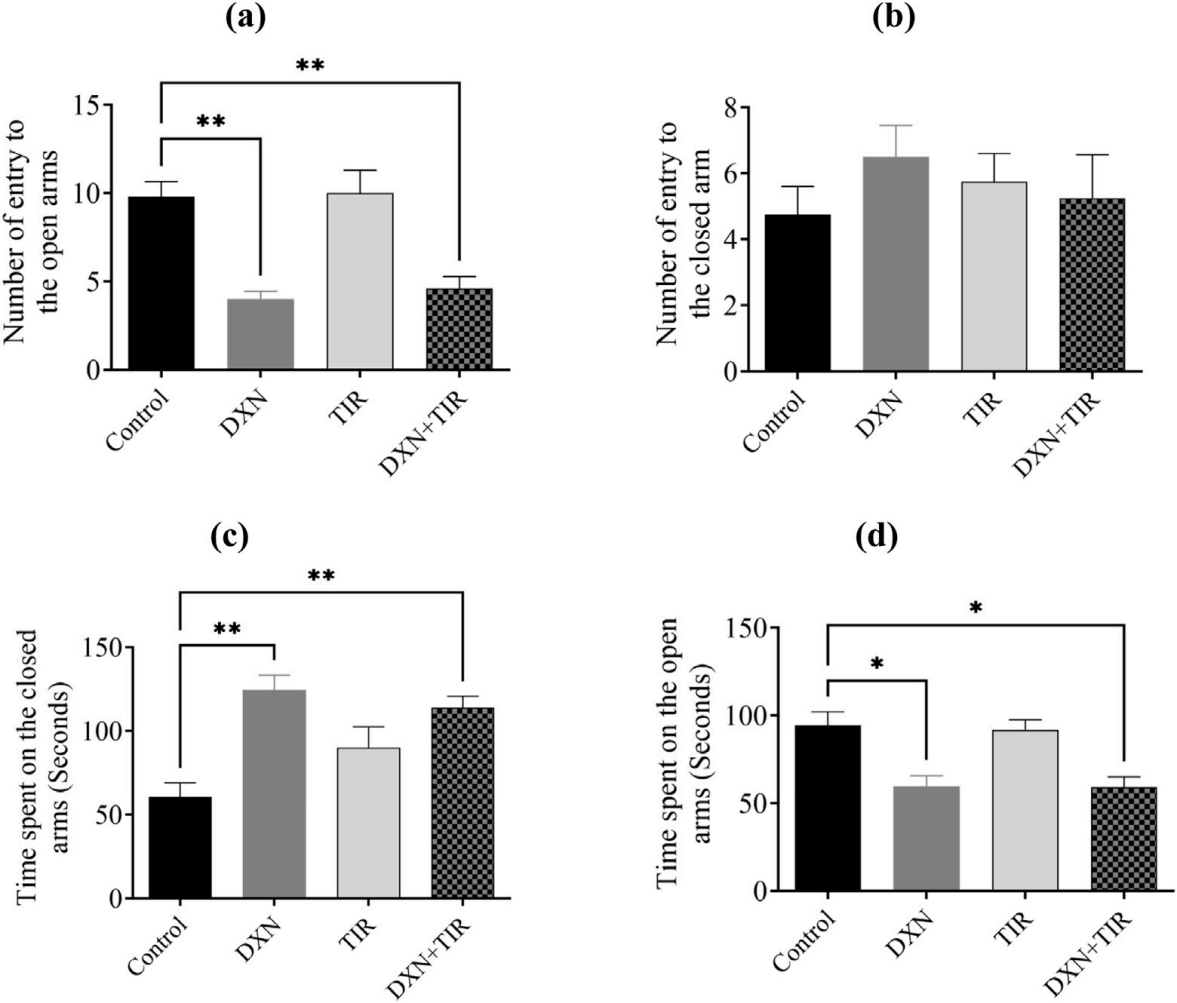
The influence of tirzepatide (TIR) on anxiety-like behavior induced by doxorubicin (DXN) was investigated by analyzing **(a,b)** the frequency of entries into, and **(c,d)** the duration spent in, the open and closed arms of the Elevated Plus Maze Test (EPMT). Changes were considered statistically significant at *p < 0.05 and **p < 0.01 when related to the control group. (n = 6).

Statistical analysis revealed significantly fewer entries into the open arms for rats in the DXN and DXN + TIR groups (*p* < 0.01) (Figure 5a). Conversely, the number of entries into the closed arms remained statistically unchanged (Figure 5b). The duration of time spent in the closed arms was pointedly longer for rats in the DXN and DXN + TIR groups (*p* < 0.01) (Figure 5c). In contrast, the time spent in the open arms was pointedly reduced in the DXN- and DXN + TIR-treated groups related to controls (*p* < 0.05), while the TIR group exhibited no significant changes (Figure 5d).

### 3.6 Effect of DXN and TIR on antioxidant enzymes in the rat brain

**FIGURE 6:**
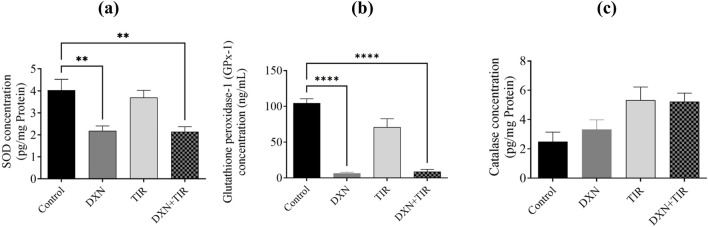
The investigation focuses on the impact of doxorubicin (DXN) and tirzepatide (TIR) on the concentrations of **(a)** SOD, **(b)** GPx, and **(c)** catalase within the rat brain. The data are presented as mean ± standard error of the mean (SEM). Group modifications were assessed using an analysis of variance (ANOVA) test. Differences were considered substantial at * *p* < 0.01 and ****p < 0.0001 related to the Control group. (n = 6).

In the group treated with DXN, the relative expression levels of SOD and GPx were significantly reduced correlated to the control group, with *p*-values of less than 0.01 and 0.0001, respectively. Conversely, the group receiving TIR did not demonstrate any significant differences from the control group. When TIR was co-administered with DXN, SOD and GPx levels remained unaffected. Additionally, catalase concentrations did not show significant changes in the groups treated with DXN, TIR, or their combination (Figure 6).

### 3.7 Effect of DXN and TIR on ROS and MDA concentrations in the rat brain

**FIGURE 7:**
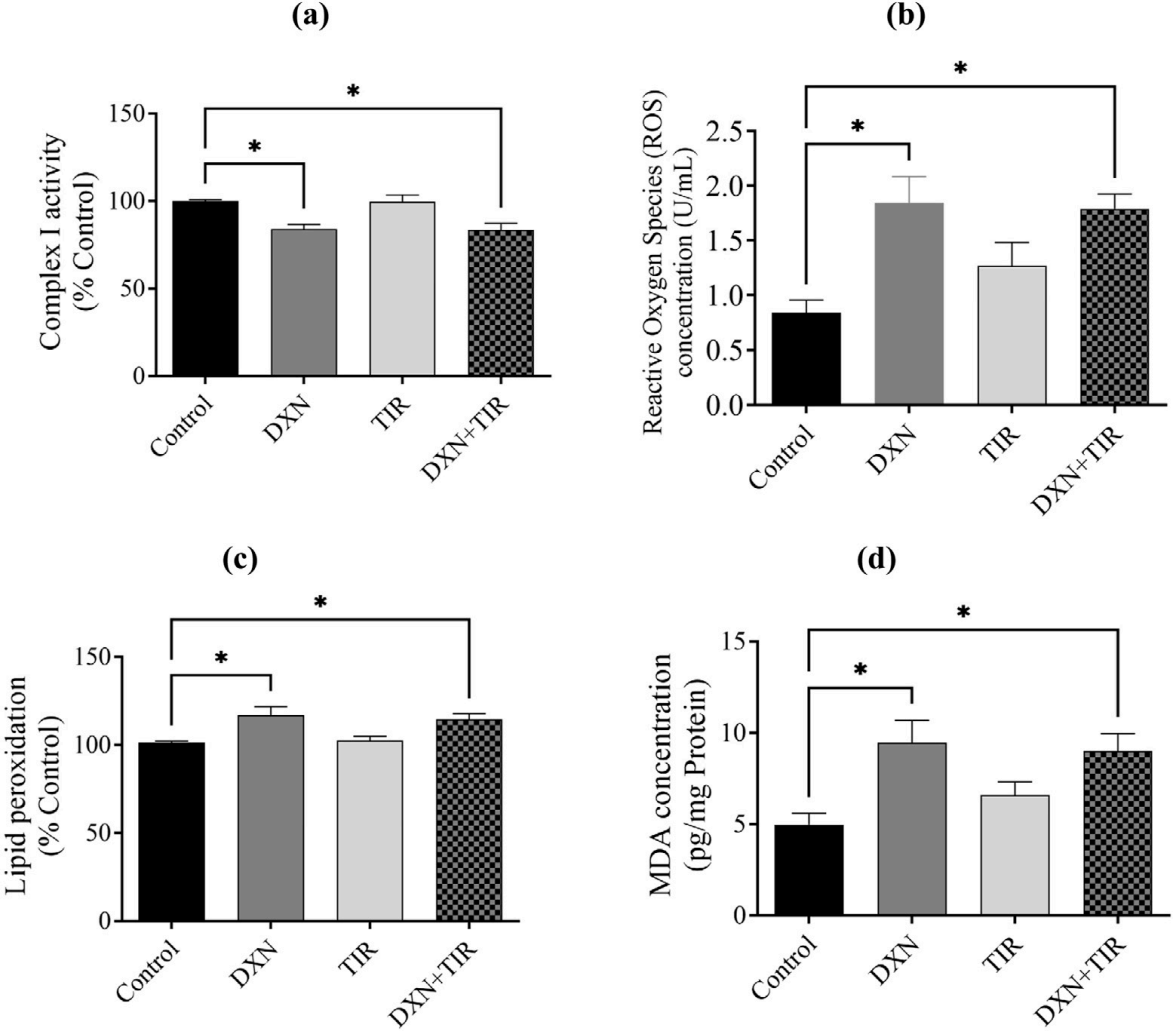
Effect of doxorubicin (DXN) and tirzepatide (TIR) on activity of **(a)** mitochondrial complex I, **(b)** reactive oxygen species (ROS), **(c)** lipid peroxidation, and **(d)** malondialdehyde (MDA) concentrations in the rat brain. Data are characterized as mean ± SEM. Group comparisons were analyzed via an ANOVA test. Alterations were considered statistically substantial at *p < 0.05 compared with the Control group. (n = 6).

The activity of mitochondrial complex I was compromised following DXN treatment, and this impairment was not ameliorated by the co-administration of TIR (Figure 7a). Moreover, ROS, lipid peroxidation, and MDA levels in the DXN were significantly elevated related to the control group (*p* < 0.05), whereas the TIR administered group showed no enhancement in either ROS, lipid peroxidation, or MDA. In the combined group TIR and DXN, TIR was unable to reverse the DXN effect by elevation of either ROS, lipid peroxidation, and MDA levels (Figures 7b–d).

## 4 Discussion

The study evaluated the potential of tirzepatide (TIR) to counteract cognitive impairment induced by DXN in a chemobrain rat model, with a particular focus on its ability to inhibit the oxidative stress pathway. DXN-induced chemobrain is known to result in significant changes in oxidative stress biomarkers in the brain, including elevated levels of ROS, lipid peroxidation, and MDA, and reduction in endogenous antioxidants such as SOD and GPx as well as mitochondrial function (Alhowail, 2024). The use of DXN to induce chemobrain in rats is highly relevant to clinical comorbid conditions, as it replicates the increased oxidative stress observed in such scenarios (Chiang et al., 2023). This model serves as a valuable tool for examining the effects of TIR in a clinically relevant context. Our findings reveal that TIR did not ameliorate the cognitive dysfunction and oxidative stress caused by DXN, indicating its lack of efficacy in protecting against oxidative stress-induced neurotoxicity in chemobrain.

The mortality rate serves as a vital metric in evaluating the risk associated with drug toxicity. Recent investigations have highlighted a link between the administration of DXN and an elevated mortality rate in specific patient groups. Prior studies have confirmed a relationship between the dosage of DXN and increased toxicity, which in turn raises the risk of mortality. The study findings revealed a 30% increase in mortality following DXN treatment, whereas the combination of DXN and TIR resulted in a 40% mortality rate after 10 days in the animal subjects, with the control and TIR groups showing no mortality. This observation is consistent with earlier findings on DXN toxicity (Alsaud et al., 2023). Additionally, the influence of DXN on body weight is indicative of its toxic effects. DXN may adversely affect organs such as the heart, liver, and kidneys, potentially leading to a decrease in body weight (Linders et al., 2024). The results demonstrated a significant reduction in body weight after 10 days of DXN treatment of approximately 18%. However, co-administration with TIR synergized the DXN effect on body weight reduction to approximately 21%. Furthermore, TIR alone was also associated with a slight decrease in body weight linked to the control group. The observed reduction in body weight following DXN and TIR treatment corresponds with a simultaneous decrease in food intake. This correlation suggests that the primary mechanism behind the weight loss is a reduction in dietary consumption. Consequently, the findings indicate that anorexia induced by DXN or TIR plays a crucial role in the observed decrease in body mass.

To ascertain the occurrence of cognitive impairment in rats administered with DXN, several behavioral assessments were conducted to evaluate hippocampal-dependent pathways involved in short-term memory encoding. These assessments included the Y-maze, NORT, and EPM, which serve as distinct indicators reliant on hippocampal function (Mani et al., 2022). Given that DXN treatment can affect overall brain function, it is anticipated to alter behavioral performance as a reflection of these changes. In the Y-maze task, DXN-treated rats exhibited a reduced sum of entries into the novel arm compared to the control group. Although the time spent in the novel arm did not differ significantly, this suggests a degree of cognitive impairment in the DXN group, in contrast to the control. The addition of TIR to DXN treatment did not reverse these changes. To ensure that the reduced number of entries was not due to lethargy, the total sum of entries was calculated and found to be not significantly different across all groups. In the NORT, DXN-treated rats spent less time exploring the novel object linked to controls. Co-administration of TIR with DXN did not mitigate this reduction in exploration time. Consequently, this test established the cognitive impairment induced by DXN and the inability of TIR to ameliorate it. The EPM task, which involves the amygdala-hippocampal pathway, revealed that DXN-treated rats made significantly fewer entries into the open arms and spent more time in the closed arms, demonstrating augmented anxiety-like behavior compared to control rats. Although TIR administration did not alter the sum of entries into open or closed arms, TIR co-treatment also failed to improve the sum of entries into the open arms or the interval of time spent in the closed arms, indicating its ineffectiveness in reducing anxiety-like behavior induced by DXN. Collectively, these tests confirmed that DXN induces cognitive impairment, consistent with previous studies (Alhowail and Aldubayan, 2023; Alsaud et al., 2023). However, our findings indicate that TIR co-treatment fails to ameliorate the cognitive deficits caused by DXN. While TIR has demonstrated efficacy in mitigating cognitive impairments associated with diabetes and in reducing cardiotoxicity induced by DXN, it has not been effective in addressing cognitive dysfunction specifically resulting from DXN exposure (Guo et al., 2023; Yang et al., 2025).

Extensive research has demonstrated a link between oxidative stress and cognitive decline (Franzoni et al., 2021; Kandlur et al., 2020). Oxidative stress arises from an imbalance between free radicals, particularly reactive oxygen species (ROS), and the body’s mechanisms for detoxifying these radicals or repairing the damage they cause. These free radicals, which are byproducts of mitochondrial damage, can harm DNA, proteins, and lipids, thereby impairing cellular function and resulting in the production of malondialdehyde (MDA). High concentrations of ROS, such as superoxide anion, are converted into hydrogen peroxide (H_2_O_2_) by the endogenous antioxidant enzyme superoxide dismutase (SOD). This hydrogen peroxide is then further reduced to water by the action of glutathione peroxidase (GPx) and catalase. Thus, it is well-established that increased ROS and MDA levels, or reduced levels of antioxidants like SOD, GPx, and catalase, signify mitochondrial dysfunction and oxidative stress, which is also associated with cognitive impairment. The association between mitochondrial dysfunction, particularly the activity of complex I, and increased levels of reactive oxygen species (ROS) has been correlated (Wen et al., 2025). Our findings demonstrated that impairment in mitochondrial complex I activity by reducing oxidized NADH concentration causes an elevation in the levels of ROS in rats treated with DXN, alongside an increase in lipid peroxidation and its product malondialdehyde (MDA) levels, confirming the occurrence of oxidative stress. Furthermore, superoxide dismutase (SOD) and glutathione peroxidase (GPx) were pointedly reduced following DXN therapy, while catalase levels remained unchanged. Although region-specific analysis of catalase activity in discrete brain areas offers insights into localized oxidative stress, measurement in whole brain homogenate provides an integrative overview of the general antioxidant status of the brain. The levels of catalase were assessed in both the cortex and hippocampus, yielding results consistent with those observed in whole brain tissues. The combination of TIR with DXN did not alter the effects of DXN on the elevation of oxidative stress biomarkers or the reduction of antioxidant levels. Therefore, the results indicate that the elevation of ROS damages neurons in the brain, leading to a loss of functionality, as evidenced by the increased lipid peroxidation and MDA levels. Additionally, the inability of SOD to neutralize the superoxide anion, which oxidizes membrane bilayers, results in cellular damage. We observed a reduction in GPx levels, while catalase levels remained unchanged. This disparity led to the accumulation of hydrogen peroxide (H_2_O_2_), as evidenced by heightened lipid peroxidation and diminished mitochondrial complex-I activity, ultimately inducing apoptosis. Although glucagon-like peptide-1 (GLP-1) activation is reported to ameliorate oxidative stress by increasing antioxidant levels, the administration of TIR with DXN did not significantly mitigate the effects of DXN on induced cognitive impairment and oxidative stress. The mitochondrial respiration and cellular energetics are regulated by inhibitory activity of GSK3β and stimulatory activity of AKT. Further, the PI3K/Akt/GSK-3β signaling pathway regulates mitochondrial complex I activity, which is essential for mitochondrial function, energy metabolism, and redox balance (Li et al., 2013). Dysregulation of this signaling pathway can decrease complex I functioning, resulting in increased production of reactive oxygen species (ROS), mitochondrial malfunction, and eventually dementia (Rai et al., 2019). Notwithstanding, TIR alleviated memory impairment in STZ-induced diabetic rats by modulating insulin resistance and the inflammatory response through restoration of the PI3K/Akt/GSK-3β signaling pathway (Guo et al., 2023).

This study possesses distinct strengths and limitations. To our familiarity, this represents the first investigation into the effects of DXN on oxidative stress alongside the possible protective benefits of TIR. Strengths include the use of a homogenous rat population (same strain, age, and weight, and confirmed cancer-free) to isolate the impact of DXN, thereby minimizing confounding variables. Furthermore, the study employed commercially sourced, hospital-dispensed drugs at multiple doses to ensure response consistency. The limitations of this study include the limited efficacy of TIR in mitigating DXN-induced mortality, weight loss, and oxidative stress, as well as the absence of histological validation for the neurological observations, particularly within the cortex and hippocampus. Our laboratory’s current technical and resource limitations precluded us from performing comprehensive histological analyses to substantiate our functional and behavioral findings. Future research should incorporate histological assessments of neuronal architecture, cellular integrity, and potential pathological alterations in these brain regions to confirm and expand upon our results. Moreover, further exploration is necessary to clarify the mechanisms underlying neurotoxicity by examining neuroinflammation and neuronal apoptosis.

## 5 Conclusion

This study reveals that DXN treatment results in an augmented mortality rate and a drop in body weight, which in turn contributes to memory impairment as evaluated through behavioral assessments such as the Y-maze, NOR, and EPM. The underlying mechanism is linked to heightened oxidative stress, as indicated by elevated levels of ROS and MDA, alongside decreased concentrations of SOD and GPx in the brain. While TIR offers a slight improvement in these adverse effects, its co-administration with DXN does not significantly counteract them. Therefore, TIR is considered an ineffective strategy for addressing DXN-induced cognitive deficits and oxidative stress.

## Data Availability

The original contributions presented in the study are included in the article/supplementary material, further inquiries can be directed to the corresponding author.
